# Book Review: Epigenetics: Current Research and Emerging Trends

**DOI:** 10.3389/fgene.2015.00347

**Published:** 2015-12-16

**Authors:** Hollie J. Rowlands, Sanna Abbasi, Krassimir Yankulov, Brandon A. Wyse

**Affiliations:** Department of Molecular and Cellular Biology, University of GuelphGuelph, ON, Canada

**Keywords:** epigenetics, DNA methylation, histones, Rett Syndrome (RTT), FSHD muscular dystrophy, book review, epigenomics, biomarkers

Epigenetics is the science of heritability, which is transmitted through chromatin structure rather than the sequence of DNA *per se.* Epigenetics also includes the broader areas of nucleosome/chromatin biology, histone modifications, and DNA methylation. In recent years, these fields of research have enjoyed massive developments. Today, we have a better understanding of the basic principles of such inheritance as well as its relevance to health disorders and disease.

*Epigenetics: Current Research and Emerging Trends* (2015, ed. Chadwick) is published at a time when the concepts first introduced in fundamental research are being applied to a breadth of new and emerging fields. This book explores several specific advances in these fields, without fixating on the historical context of the research. The title is a true representation of the content the reader will discover. The book has incorporated three broad themes. The first one deals with select fundamental epigenetic processes including nucleosome dynamics, histone chaperones, long non-coding RNA, DNA methylation, sex chromosome-dosage compensation, and centromere function. This theme covers a range of fields in significant and very specific detail without unifying the diverse scope of articles or attempting to provide comprehensive knowledge on epigenetics. The other two themes are epigenetics of human disorders and cancer and the link between the epigenome and the environment. Again, the emphasis is on specific topics rather than the breadth of the specific field.

The book consists of 17 chapters written in a literature review style. Each is written either by a single author or a group of researchers who are experts in their respective fields. The editor, Chadwick, contributed to three of the 17 chapters. Some of the chapters also contain a limited amount of data and/or tables that summarize and enhance the key messages from the text. The chapters include an abstract, an introductory section, and numerous detail-rich subsections followed by a conclusion and future research directions. This formatting is easy to navigate and well organized; abstracts and sub-headings allow the reader to select chapters and sub-sections of interest for further reading. Current methods in epigenetic research are highlighted alongside the detailed models of epigenetic processes allowing the reader to become well versed on current techniques and findings. In addition, thorough referencing throughout the chapters and the list of additional resources (including recommended textbooks and review articles) provide many opportunities for further research about these and other topics in epigenetics. For these reasons, we find that this book would serve as a great resource for seasoned researchers or incoming graduate students to get a solid representation of current work in the field. They can also be used as case study reports for upper year undergraduate students in a course on epigenetics.

The first eight chapters provide advanced knowledge on basic epigenetic processes as derived from model organisms. In chapters 1–4, we learn about recent developments in DNA methylation, histone modifications, and the activities of sirtuin proteins. In chapters 5 through 8, these same concepts are presented in the context of their effects on the regulation of the genome and gene expression.

The remainder of the book is a diverse selection of topics including human diseases, diagnosis of epigenetic disorders, emerging ideas in epigenetics, and the role of the environment, and metabolism in epigenetics. Three chapters are dedicated to epigenetic effects in human diseases such as Rett syndrome (RTT), Facio-Scapulo-Humeral Dystrophy (FSHD), and cancer. Each chapter demonstrates that de-regulation of the epigenome can lead to the onset or to the increase in the severity of such disorders. In contrast to the rest of the book, these chapters include a clinical perspective and outline potential treatment options. Of these chapters, chapter 14 places a greater emphasis on clinical application by proposing the use of epigenetic biomarkers for cancer diagnosis, prognosis, and treatment. These three chapters offer a unique medical viewpoint and are a valuable resource to researchers and medical practitioners alike.

Another three chapters highlight some emerging ideas and exciting new lines of research in the field of epigenetics. Each of these chapters begins with solid background information before delving into the main topic. The “hot topics” include long non-coding RNA, the epigenetics behind reprogramming cells to pluripotency, and guanine-quadruplex DNA secondary structures (G4 motifs). Each chapter provides a strong, comprehensive analysis of emerging directions of epigenetic research.

The final trio of chapters in the book focuses on the relationship between epigenetics and the environment. The content of each chapter is based on the understanding that gene-environment interactions are transmitted through changes to the epigenome. These chapters look at the effects of various environmental factors, including toxins, stress, nutrition, and diet. From all of these chapters, we learn that environmental factors can have a substantial and long-lasting impact on human health and can result in increased susceptibility to various diseases. The chapters as a whole bring attention to the important role played by the environment and provide a broad introduction for readers new to the topic.

In conclusion, this book provides an in-depth analysis of many topics in epigenetics. Using extensive information of both model organisms and applications in medical genetics, the authors present contemporary cutting-edge research and techniques in an easy-to-read format. The depth of the content is suitable for an expert in the field and is also an appropriate resource for newer researchers who are looking to expand their knowledge about epigenetic concepts.

At times, the order of the chapters is eclectic and would have benefited from an introductory section to unify the underlying themes. However, this deficiency did not impact the readability of what is clearly an all-encompassing book of epigenetics. On the other hand, the true value of this volume is its total dedication to recent developments and cutting-edge research. We strongly recommend this book to any reader who is looking to enhance their knowledge on modern epigenetics.

## Author contributions

All authors contributed equally in the review and writing of this manuscript. The writing of this review was funded by a Natural Sciences and Engineering Research Council of Canada Grant (Grant# 400478).

### Conflict of interest statement

The authors declare that the research was conducted in the absence of any commercial or financial relationships that could be construed as a potential conflict of interest.

